# Fake news? The impact of information mismatch on mating behaviour

**DOI:** 10.1002/ece3.11493

**Published:** 2024-07-17

**Authors:** Leonor R. Rodrigues, Sara Magalhães

**Affiliations:** ^1^ cE3c: Centre for Ecology, Evolution, and Environmental Changes, Faculty of Sciences & CHANGE ‐ Global Change and Sustainability Institute University of Lisbon Lisbon Portugal

**Keywords:** first male sperm precedence, mate choice, mating costs, reproductive behaviour, spider mites

## Abstract

Multiple cues are often used for mate choice in complex environments, potentially entailing mismatches in the information conveyed by different sources. We address the consequences of this information mismatch for receivers using the spider mite *Tetranychus urticae*, in which virgin females are highly valuable mates compared to mated females, given first male sperm precedence. Accordingly, males are known to prefer virgins and distinguish them using cues from the females themselves and that they leave on the substrate. Whereas cues from females are highly reliable, those left on the substrate may not reflect the real female mating status if females move and/or mate. Here, we tested the consequences of such mismatch by exposing males to mated or virgin females on patches previously impregnated with cues deposited by females of either mating status. Male mating attempts were solely affected by substrate cues while female acceptance and the number of mating events were independently affected by both cues. Copulation duration, in contrast, depended mainly on the mating status of the female, with the number of copulations and the total time spent mating being intermediate in environments with mismatched information. We also show that males incur mating costs, reflected in reduced survival in environments with virgin cues. These results suggest that substrate cues left by females are instrumental for males to find their mates. However, in environments with mismatched information, males may pay survival costs without the associated benefit of mating with virgins, or they may lose opportunities to mate with virgins by responding to substrate cues from mated females. The benefit of using multiple cues will then hinge upon the frequency of information mismatch, which itself should vary with the dynamics of populations.

## Graphical abstract

Mismatches between cues can occur when they persist in the environment for different periods of time, potentially increasing uncertainty about which mate to choose. We found that patches with a mismatch between cues signalling female mating status led to intermediate survival costs in males, with the substrate cues left by females being instrumental for males to find their mates quicker but at the expense of mating with females and not fertilising their offspring.

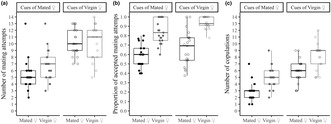

## 1 INTRODUCTION

In many taxa, individuals use multiple sources of information to search for mates and engage in copulations (Bretman et al., 2011; Bro‐Jørgensen, 2010; Candolin, 2003; Coss et al., 2022; Dore et al., 2018; Hebets & Papaj, 2005; Jennions & Petrie, 2007; Ronald et al., 2020). Several hypotheses have been put forward to explain the role of different cues in mate decision (see detailed classifications in Bro‐Jørgensen, 2010; Candolin, 2003; Hebets & Papaj, 2005; Jennions & Petrie, 2007): (a) each cue may convey a piece of information about different mate qualities, the cues together increasing the accuracy of assessment; (b) cues can be redundant but together improve discrimination, reducing errors associated with each cue; and/or (c) cues can work differently in distinct environments and/or at dissimilar distances. The use of multiple cues is particularly useful in varying and complex environments (Bro‐Jørgensen, 2010; Dore et al., 2018) where a disruption in the transfer of information, via excess noise or a mismatch between cues, is more likely, as it may contribute to an accurate and fast response across environmental conditions (Candolin, 2003, 2019; Coss et al., 2022; Dore et al., 2018).

Mismatches among cues can occur when they have different susceptibilities to changes in the environment, or when they persist for different periods of time (Bro‐Jørgensen, 2010; Fawcett & Frankenhuis, 2015). For instance, during development, the butterfly *Pieris rapae* relies on both temperature and the photoperiod to evaluate climatic conditions at adult emergence. Climate warming leads to a mismatch between these two cues, affecting temperature but not the photoperiod, which can result in a sub‐optimal wing melanisation phenotype upon emergence (Stoehr & Wojan, 2016). Likewise, mate quality can be perceived via both ephemeral or plastic cues (e.g. behavioural traits), and more permanent cues such as morphological traits (Bro‐Jørgensen, 2010). That is the case of the field cricket (*Gryllus campestris*), which uses both body size and chirp rate in males as indicators of mate quality, typically giving priority to body size, the most permanent cue (Scheuber et al., 2004). In variable environments, however, fixed cues may become unreliable indicators of male quality (Higginson & Reader, 2009), in which case the use of ephemeral cues may be favoured.

Whereas the use of multiple cues can be beneficial to receivers in many contexts, their use also comes at some costs. First, processing information stemming from multiple cues is expected to lead to increased energetic and cognitive investment (Schneeberger & Taborsky, 2020). Second, when there is information mismatch, using multiple cues may lead to an inaccurate response. When this perception of cues concerns mate choice, costs for receivers are likely to be associated with missing opportunities of mating with a suitable mate or with investing in matings with unsuitable mates (Muñoz & Blumstein, 2012). For example, the presence of heterospecific female stimuli compromises conspecific chemical cue detection by male *Gryllus integer* crickets, leading to equal intensity of heterospecific and conspecific courting (Leonard & Hedrick, 2009). Therefore, the optimal use of multiple cues and corresponding mating behaviour should depend on the balance between the costs of acceptance and rejection errors (Scharf et al., 2020).

The existence of cue mismatch in mate choice can be particularly disadvantageous in species with first male sperm precedence, where female mating status discrimination is essential for male mating success. Indeed, under this pattern of sperm precedence, mating with mated females provides low, if any, fertilisation opportunities, whereas mating with virgin females strongly contributes to reproductive success (Thomas, 2011). Accordingly, males of species with first male sperm precedence have the ability to discriminate female mating status, preferring the virgins, and modulate their reproductive behaviour based on the cues presented by females (Rodrigues et al., 2017; Rypstra et al., 2009; Stoltz et al., 2007; Thomas, 2011; Yasui, 1994). Yet, how males respond to discordant information concerning the female mating status and how such putative shift in behaviour translates into mating costs remain largely unknown.

To fill this gap, we observed the mating behaviour of male and female two‐spotted spider mites (*Tetranychus urticae*) in environments with information concerning the female mating status coming from two sources, the females themselves and the cues they leave in the substrate. Spider mites have first‐male sperm precedence (Helle, 1967; Rodrigues et al., 2020), with the first males mating with a female siring at least 95% of the female's offspring (Rodrigues et al., 2020). Accordingly, males prefer to mate with virgins, basing their decision upon cues that the females leave on the substrate and/or volatiles released by females (Oku, 2010; Rodrigues et al., 2017). Furthermore, matings with virgin females take less time to start and are longer than matings with mated females, inducing more survival costs in males (Oku, 2010; Rodrigues et al., 2017, 2020). All this suggests that male reproductive investment in matings with virgins or with mated females is not the same. However, matings involving mated females are frequently observed, even in the presence of virgins (Clemente et al., 2016; Oku, 2010), despite often leading to lower fecundity (Macke et al., 2012; Rodrigues et al., 2020). Possibly, discrimination in this species is not perfect and may depend on the composition of cues present in the environment. Spider mite populations occur in variable environments, as they colonise seasonal resources such as agricultural crops (Helle & Sabelis, 1985). Moreover, they disperse among patches after a variable number of generations in the same patch, (i.e. they follow a subdivided haystack population structure; Nagelkerke & Sabelis, 1996; Smith, 1964). Indeed, new patches (i.e. plants) are generally colonised by one or few mated females, which will initially lay eggs that will reach adulthood synchronously. Then, mites will remain on the colonised patch for some generations, which will progressively become less discrete until the plant is over‐exploited, and then dispersal will happen again. Hence, a concordance between substrate and contact cues is expected in the initial phase of colonisation, with a mismatch building up with patch age. Here, we tested the consequences of information mismatch within this context. We predict that, in an environment with information mismatches, the chance of mating with less favourable females and losing valuable mating opportunities is higher than in environments with concordant information, which should influence the overall mating costs suffered by males.

## 2 MATERIALS AND METHODS

### 2.1 Spider mite populations and rearing conditions

The spider mite population used was created from an outbred population of *Tetranychus urticae*, established in 2016 at the host laboratory, by merging six populations collected in the field around Lisbon in 2013 (Rodrigues et al., 2022; Zélé et al., 2018). The population was reared in large numbers (>200) on bean plants (*Phaseolus vulgaris*, Fabaceae, var. Contender; Germisem Sementes Lda, Oliveira do Hospital, Portugal), under controlled conditions (25°C, photoperiod of 16 L: 8D). All bean plants used in the experiment were grown for 14 days in a herbivore‐free climatic chamber, under the same controlled conditions as spider mites.

### 2.2 Experimental setup

All experiments were conducted on bean leaves. Females and males were isolated from the base population on detached leaves at the quiescent stage, immediately before completing the last moult. At this stage, both sexes are immobile and females are bigger and rounder than males, which facilitates sexing. This way, all individuals used in the experiment shared the same age at maturity, and virginity was ensured in both sexes before they were allocated to different treatments.

Males of this species use volatiles emitted by females and substrate cues left by females on patches to choose between virgin and mated female male spiders (Rodrigues et al., 2017). Based on this information, we created conditions for mate discrimination to take place in an environment in which there was matching information (i.e. virgin females on patches previously impregnated with cues released by virgin females or mated females on patches previously impregnated with cues released by mated females) or in which there was a mismatch between the information released by the females present on the patch and the cues that were left on the substrate by previous females (i.e. virgin females on patches previously impregnated with cues released by mated females or mated females on patches previously impregnated with cues released by virgin females).

To create these different environments, multiple groups of 10 virgin females were randomly assigned to patches (leaf discs of 2.55 cm^2^) with three virgin males. Behaviour was observed for 1 h and, when a mating occurred, the mated female was transferred to a new empty patch of the same size (up until the 10 females were moved into the new patch). Simultaneously, groups of 10 virgin females were directly transferred to similar empty patches without ever being in contact with males. Both types of females were left on those patches for 24 h such that they could release cues that remained on the substrate. Those females were then removed, and five new females (either mated or virgin) were placed on those patches. Subsequently, one focal male was added to all patches. Henceforth, for simplicity, we refer to the cues left on the patch by virgins or by mated females that were removed prior to the beginning of the mating sessions as ‘substrate cues’ and the information emitted by females present on the patch (including their own behaviour) as ‘female mating status’. Note that, although we are aware that chemical information is sufficient for mate choice (Rodrigues et al., 2017), it is possible that other cues or signals (e.g. tactile and visual) are used by males as well (Royalty et al., 1993). In any case, we assume that the information provided by the presence of a female is composed of multiple cues that are concordant, and thus, for simplicity, consider ‘female mating status’ a unit of information. Likewise, substrate cues include web, faeces, eggs and any chemical information left behind by females; once more we consider those a unit of information. We cannot rule out the possibility that new substrate cues are being deposited by the females present on the patch, but given the short duration of trials (1 h), these are not expected to play a significant role in the behaviours described.

Male and female behaviour, that is, the number of male mating attempts, the frequency of female acceptance, the number of mating events (i.e. the number of copulations) and copulation duration were observed for 1 h. A mating attempt was registered whenever a male touched the female with the two front legs and started bending its opisthosoma (Oku, 2014). Whenever a mating attempt resulted in the insertion of the male aedeagus into the female abdomen for more than 1 min, the observer registered it as the occurrence of a mating event (Satoh et al., 2001). It has been shown that females can reject male mating attempts by moving away from them (Rodrigues et al., 2020). As such, we also studied the frequency of female acceptance, calculated as the number of mating events over the number of mating attempts. Copulation duration was registered as the time in seconds a male spent with his aedeagus inside a female. Note that females were not removed from the patch during the mating session; as such, males may have mated more than once with the same female.

Subsequently, males were transferred individually to a new patch (2.55 cm^2^), made from uninfested bean plants, and their survival was followed daily to measure whether different mating histories would translate into a longevity cost. Death was classified as natural (i.e. the corpse was found on the patch) or censored (i.e. males died by drowning or by being accidentally stuck in the leaf or squeezed).

This experiment was carried out in 21 mating sessions divided into 8 days. In total, 84 males and 420 females were observed, corresponding to 21 males and 105 females per treatment (i.e. every combination of substrate cue and female mating status).

### 2.3 Statistical analyses

All analyses were carried out using the R statistical package (v. 3.5.2) and can be reproduced using the data and code publicly available in Dryad (Rodrigues & Magalhães, 2024). The same model structure was followed for the analysis of all traits: the substrate cues (i.e. cues left on the patch by virgins or by mated females that were removed prior to the beginning of the mating sessions) and the female mating status (i.e. virgin or mated females present on the patch during the mating session) were fitted as fixed explanatory variables, whereas block (the day and time of the day at which the experiment was done) was fitted as a random explanatory variable (see Table S1).

Copulation duration was examined as ‘copulation duration of the first mating’ and ‘copulation duration across mating events’. In the analysis of the latter variable, the order of each copulation (i.e. whether it was the first, second, third mating, etc.) was added as a covariate. All possible interactions between fixed factors were included.

The number of mating attempts and the number of mating events (i.e. the number of copulations) were analysed using a Poisson distribution (*glmer*, lme4 package; Bates et al., 2015). The frequency of female acceptance (the number of female acceptances over the number of female rejections and acceptances) was analysed using a binomial distribution (*glmer*, lme4 package; the formulation of the dependent variable including the number of female rejections and acceptances within a *cbind* function). The duration of the first mating and the copulation duration across events were tested for normality and analysed using linear mixed‐effect models (*lmer*, lme4 package; Bates et al., 2015). Male survival was analysed using a Cox proportional hazards mixed‐effect model (*coxme*, coxme package; Therneau, 2015), with the death of males being classified as natural or censored. This last model included the number of matings as a covariate.

All maximal models were simplified by sequentially eliminating non‐significant terms from the highest‐ to the simplest‐order interaction (Crawley, 2007). The significance of the explanatory variables was determined using Wald *F* tests, for continuous distributions and χ^2^ tests for discrete distributions (Bolker et al., 2009).

## 3 RESULTS

**FIGURE 1:**
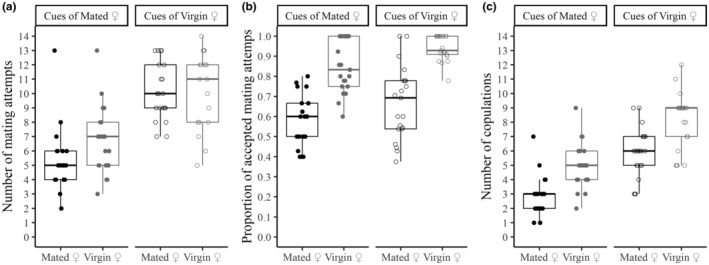
Male and female pre‐copulatory mating behaviour and the corresponding number of mating events in response to substrate cues and female mating status. Males were exposed for one hour to five virgin or mated females in patches impregnated with cues of virgin or mated females. (a) Males approached females significantly more often in patches with substrate cues of virgin females; (b) Virgins accepted male mating attempts significantly more often than mated females and the frequency of female acceptance was higher in patches with cues of virgin females; (c) The number of mating events was significantly higher when males mated with virgin females and when they occurred on patches with cues of virgins. Circles represent individual replicates. Black circles – patches with mated females; grey circles – patches with virgin females; open circles – patches with cues of virgin females; full circles – patches with cues of mated females.

Results from all statistical analyses are presented in Table S2. Males approached females more often in patches with substrate cues of virgin females, independently of the mating status of the females present on the patch (substrate cues x mating status: χ^2^_1_ = 1.104, *p* = .293; patch cues: χ^2^_1_ = 54.323, *p* < .01; mating status: χ^2^_1_ = 2.055, *p* = .152; Figure 1a). Virgins accepted male mating attempts more often than mated females and the frequency of female acceptance was higher in patches with cues of virgin females (patch cues × mating status: χ^2^_1_ = 2.625, *p* = .105; patch cues: χ^2^_1_ = 8.553, *p* < .01; mating status: χ^2^_1_ = 64.252, *p* < .01; Figure 1b). The number of mating events (i.e. the number of copulations) was also affected by both the substrate cues and the mating status of the female independently (patch cues x mating status: χ^2^_1_ = 1.274, *p* = .259; patch cues: χ^2^_1_ = 35.445, *p* < .001; mating status: χ^2^_1_ = 17.89, *p* < .001; Figure 1c). Indeed, the number of mating events was lower when matings were with mated versus virgin females and when they occurred on patches with cues of mated versus virgin females (Figure 1c).

**FIGURE 2:**
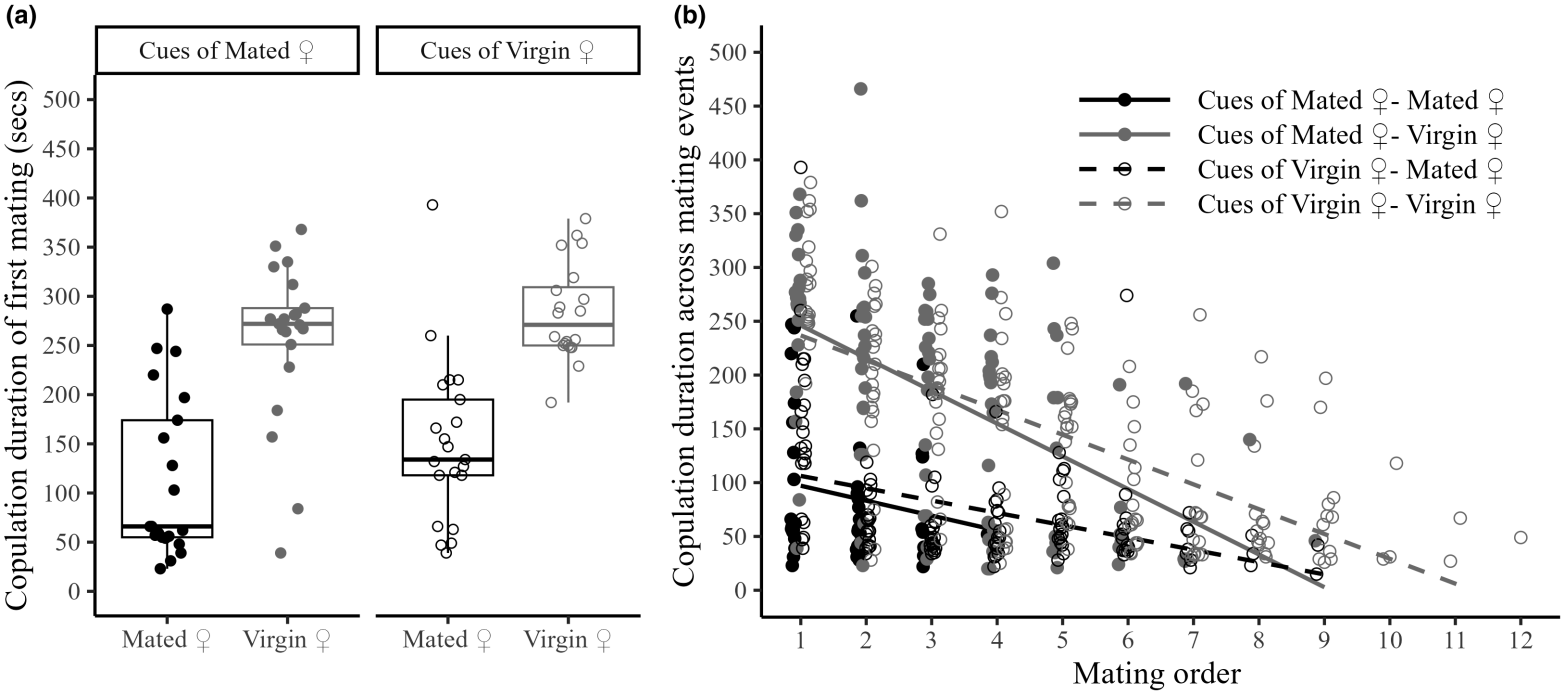
Duration in seconds (a) of first matings and (b) across mating events, in response to patch cues and female mating status. Males were exposed for one hour to five virgin or mated females on patches impregnated with cues of virgin or mated females. The first mating lasted the longest when it involved virgin females and when it occurred in patches with cues of virgins. Copulation duration significantly decreased across mating events, being this decrease steeper when males were in patches with virgin females. Circles represent individual replicates. Black circles – patches with mated females; grey circles – patches with virgin females; dashed lines and open circles – patches with cues of virgin females; continuous lines and full circles – patches with cues of mated females.

The first mating of a male lasted longer when it involved virgin females and when it occurred on patches with cues of virgins than when it involved mated females and occurred on patches with cues from mated females (patch cues x mating status: *F*_1,60_ = 0.002, *p* = .966; patch cues: *F*_1,61_ = 4.737, *p* = .033; mating status: *F*_1,61_ = 70.367, *p* < .001; Figure 2a). Copulation duration was always significantly higher in patches with cues of virgins than in patches with cues of mated females (patch cues: *F*_1,72.70_ = 4.624, *p* = .035; Figure 2b) but it significantly decreased across mating events and this decrease was steeper when males were placed on patches with virgin females (copulation order × mating status: *F*_1,364.06_ = 5.652, *p* = .018; Figure 2b).

**FIGURE 3:**
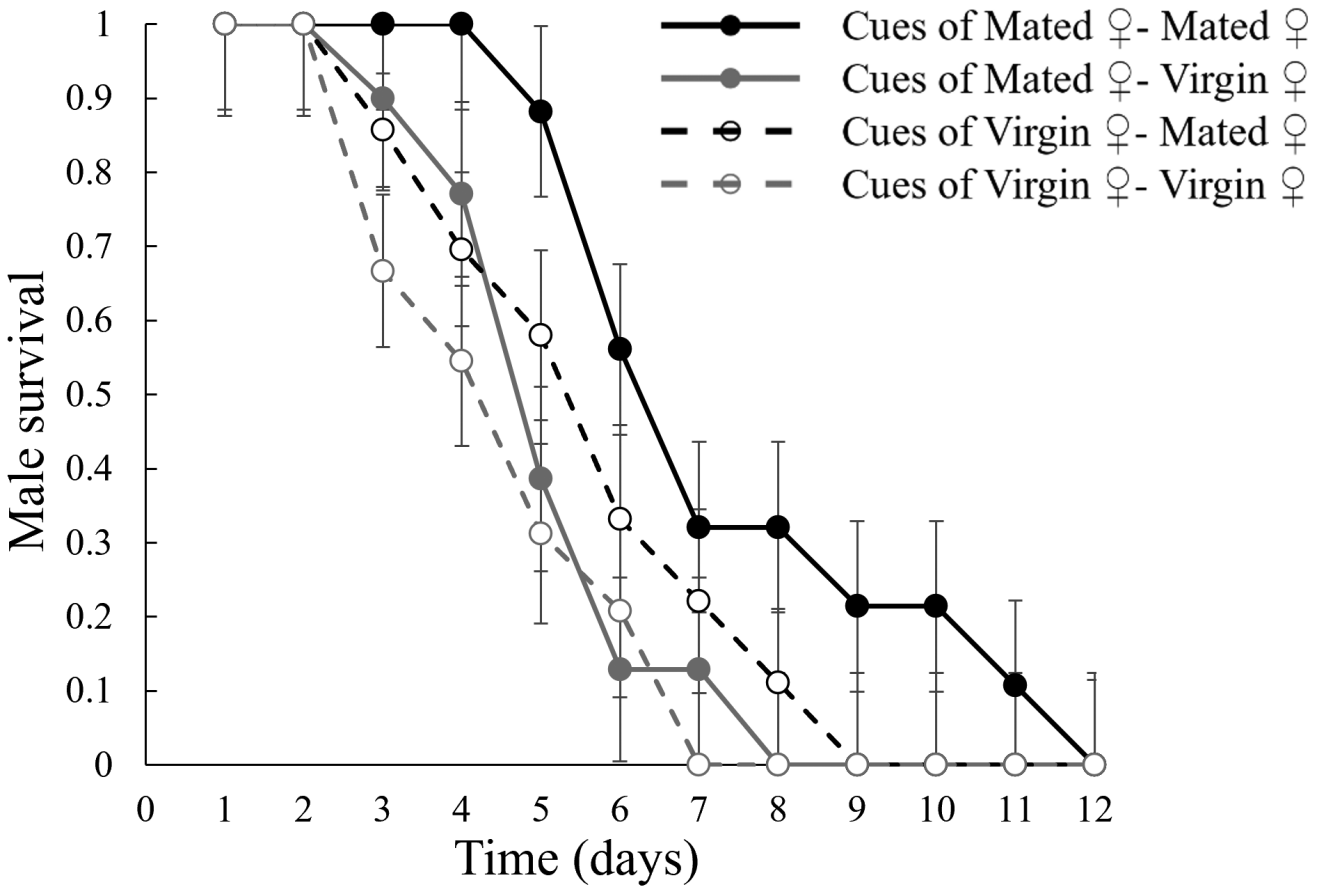
Male survival curves in response to patch cues and female mating status. Male survival was followed daily after males were exposed for one hour to five virgin or mated females on patches impregnated with cues of virgin or mated females. Male survival was significantly reduced in the presence of virgin females and on patches with cues left by virgin females compared to that of males on patches with cues left by mated females or with mated females present. Circles represent mean values per day per treatment. Black circles –patches with mated females; grey circles – patches with virgin females; dashed lines and open circles – patches with cues of virgin females; continuous lines and full circles – patches with cues of mated females. Vertical bars correspond to standard errors of the mean.

Male survival on patches with cues left by virgin females and with virgin females present was reduced compared to that of males on patches with cues left by mated females or with mated females present (patch cues x mating status: χ^2^_1_ = 0.496, *p* = .481; patch cues: χ^2^_1_ = 4.283, *p* = .038; mating status: χ^2^_1_ = 8.774, *p* = .003; Figure 3). The number of matings did not influence male survival (χ^2^_1_ = 0.051, *p* = .821).

## 4 DISCUSSION

Here, we examine the impact of multiple cues and the potential resulting information mismatch on the mating behaviour of spider mites. We found that the number of mating attempts was only influenced by cues left on the patch prior to the mating sessions, being higher in patches with cues of virgins. In turn, female acceptance was affected by both substrate cues and the mating status of the females, being the highest in patches with virgins and with substrate cues of virgin females and the lowest in patches with mated females and with substrate cues of mated females. As a consequence, the number of mating events was affected by both substrate cues and the mating status of the females, such that the number of copulations was intermediate in environments with mismatched information. Once copulation started, its duration depended mainly on the mating status of the female being fertilised, with the overall amount of time spent mating being higher in matings with virgins than with mated females and intermediate in environments with mismatched information. Male survival was the lowest in patches where all cues stemmed from virgins, intermediate in patches with mismatched information and the highest when all cues were from mated females.

In species with first male sperm precedence, like spider mites and many spiders, the sperm from the first insemination sires most of the offspring (>95% in *T. urticae*; Rodrigues et al., 2020), thus virgins are more valuable mates than mated females. Accordingly, males exhibit a preference for virgin females, in this (Oku, 2013; Rodrigues et al., 2017) and other species with the same pattern of sperm precedence (Rypstra et al., 2009; Stoltz et al., 2007; Yasui, 1994). Such result is recapitulated here in patches with concordant cues, with mating eagerness, copulation duration and survival costs being the highest in patches with all cues being from virgins.

When exposed to discordant information, males based their pre‐copulatory mating behaviour solely on the substrate cues on the patch. These cues are obviously less reliable determinants of the status of the female on those patches than information emanating from the female itself. Thus, the fact that pre‐copulatory mating behaviour is triggered by substrate cues only suggests that these cues are the most far‐reaching of the two, being beneficial during mate searching. A non‐exclusive alternative is that substrate cues are cheaper to assess, in which case it may pay off to consider them alone (Fawcett & Johnstone, 2003). For example, in the field cricket (*Gryllus integer*), calling songs are used as long‐distance cues to find mates while short‐range chemical cues are employed to assess mate quality (Leonard & Hedrick, 2010). Interestingly, being exposed to attractive long‐distance cues results in a quicker positive response to short‐distance cues, which suggests a reduced investment in the assessment of the latter cue in this species (Leonard & Hedrick, 2010). A similar pattern could be taking place here. The use of more unreliable information in pre‐copulatory mating behaviour might help explain why, in spider mites and perhaps in species with a similar pattern of sperm precedence, matings with mated females are frequently observed, despite their weak reproductive value.

Unlike male eagerness, female acceptance depended both on the substrate cues and on the females' own mating status, with increased acceptance in mated females and reduced acceptance in virgins in environments with mismatched information, compared to concordant environments. Possibly, mated females accept more matings on patches with substrate cues of virgins because those are the patches in which the number of male mating attempts is the highest and thus, resistance is expected to be more costly. This strategy, called ‘convenience polyandry’, should occur under intense harassment, when by accepting more mates than their optima, females suffer fewer costs than by resisting them (Boulton et al., 2018; Snook, 2014; Thornhill & Alcock, 1983). Such is the case, for instance, in female water striders that modify their mating rate based on the relative costs of mating and resisting mating attempts (Boulton et al., 2018). In turn, reduced acceptance of virgin females on patches with substrate cues of mated females could be a byproduct of males' reduced eagerness to mate on those patches, which may result in meek, and thus easy‐to‐reject, mating attempts. Importantly, because females' pre‐copulatory behaviour depended both on their own mating status and on the substrate cues present but that of males only relied on substrate cues, the response of the two sexes was not aligned, resulting in an intermediate number of mating events in environments with mismatched information.

Once copulation started, the response of males seemed to be more affected by the mating status of the female mating than by substrate cues present in the environment. This is probably because, once males reach the females, they have access to the more reliable cues emanating from the female itself and can thus use those to accurately modulate their post‐copulatory strategies. Still, substrate cues played a, albeit less significant, role in copulation duration. Indeed, when exposed to virgin cues, males mated for a longer period with both virgin and mated females than when exposed to cues of mated females. This suggests the information obtained before mating keeps influencing mating behaviour even when more accurate cues are available.

The response of males to multiple cues, including both pre‐ and post‐copulatory behaviours, must come at some costs. Male survival in spider mites is differentially affected by the mating status of their reproductive partners: matings with virgin females result in high offspring yield but reduced male survival, while matings with mated females lead to no offspring but also fewer survival costs (Rodrigues et al., 2020). Here, being exposed to discordant cues influenced the impact of mating on male survival. First, on patches occupied by mated females, male survival was lower when substrate cues were from virgins than when they were from mated females only. This suggests that, in the former case, males behave with mated females as they would with virgins, hence the cue mismatch leads to an over‐investment in ineffective matings. This behaviour could be maintained so as not to risk rejecting mating opportunities with suitable females, as proposed by Reeve (1989). In his model, Reeve shows that males are expected to exhibit more permissive mating acceptance thresholds as the value of the desirable female increases and the costs of accepting a wrong female decrease, which are the exact conditions we find in this system. Indeed, virgin females are highly valuable compared to mated females and the cost for males of mating with mated females is quite low (Rodrigues et al., 2020). An equivalent decrease in the acceptance threshold would be expected if assessing multiple cues was too costly, in which case one would expect individuals to neglect the least reliable cue (Muñoz & Blumstein, 2012; Schneeberger & Taborsky, 2020; Tibbetts et al., 2020), that is, the cues left on the substrate by females.

Male survival in patches with mismatches between cues is higher than in patches with cues of virgins only. In these patches, the number of mating attempts is similar to that in patches with virgins, but the total number of matings and the total amount of time spent copulating is significantly lower. This suggests that the number of mating events and/or postcopulatory events are important determinants of male mating costs, ensuring a reduction in the costs of reproduction in mating with less valuable females. Moreover, male survival was higher in patches with virgin females but substrate cues of mated females, than in patches with cues of virgins only. Therefore, it seems that in these conditions, males invest less in effective matings, possibly via a reduction in the number of male mating attempts and in the total amount of time spent mating. However, we have not tested whether the observed reduction in copulation duration is translated into reduced mating success and previous results suggest copulation duration does not correlate positively with offspring production (Satoh et al., 2001).

We did not measure the chemical composition of the different sources of information that males were exposed to, nor do we know exactly which cues are used by males to choose between virgin and mated females. Indeed, we know that substrate cues and volatiles emitted by females are sufficient for male choice (Rodrigues et al., 2017) but other cues, such as visual and tactile cues, not being necessary, may play a role (Royalty et al., 1993). In addition, substrate cues themselves, considered here as a unit of information, include web, faeces, eggs and any chemical compounds deposited by females in the patches. Web is used, among other functions, in mate searching behaviour (Penman & Cone, 1972, 1974) but the role of other substances is unknown. Still, we can make a few inferences from the patterns observed in male behaviour upon exposure. For example, we do not know whether the chemicals used by males to assess females themselves are the same as those present on the substrate. This is, however, not very likely, as different components of male mating behaviour react differently to the different combinations of cues from virgins and/or mated females. Another possibility is that only virgin females produce cues. Our results are compatible with this possibility. Regardless, this would still mean that males are exposed to situations in which the information stemming from the females themselves and the substrate they occupy are either concordant or discordant.

The optimal use of information and corresponding behaviour should depend on the balance between the costs of acceptance and rejection errors (Scharf et al., 2020) and this, in turn, should vary with the dynamics of the social and ecological environment, which set the stage for different selection pressures to operate upon mating cues and their perception (Alpedrinha et al., 2019). In spider mite populations, individuals disperse among patches after a variable number of generations in the same patch (i.e. they follow a subdivided haystack population structure; Nagelkerke & Sabelis, 1996, Smith, 1964). Such cycles of colonisation expansion foster the conditions for information mismatches within a patch. Indeed, while the information emitted by females will change simultaneously with the shift in mating status, the cues left on the patch should remain unaltered for some time after this shift. While these cues seem to be less reliable than the information provided by females themselves, they are probably accessible at a larger scale, allowing males to move in the direction of areas with suitable mates (i.e. virgins) before their competitors. This should be highly advantageous in species with first male sperm precedence. These findings could thus have important implications for mating system evolution, potentially helping to explain why female multiple mating is maintained in species with first male sperm precedence. Still, the benefit of using multiple, sometimes discordant, cues will hinge upon the frequency of discordance among them, which itself should vary with the dynamics of populations.

## AUTHOR CONTRIBUTIONS

**Leonor R. Rodrigues:** Conceptualization (equal); data curation (lead); formal analysis (lead); funding acquisition (equal); investigation (equal); methodology (equal); project administration (equal); resources (equal); validation (equal); visualization (lead); writing – original draft (lead); writing – review and editing (equal). **Sara Magalhães:** Conceptualization (equal); formal analysis (supporting); funding acquisition (equal); investigation (equal); methodology (equal); project administration (equal); resources (equal); validation (equal); writing – original draft (supporting); writing – review and editing (equal).

## FUNDING INFORMATION

This work was supported by the European Research Council (grant number COMPCON, GA725419) to SM and the Fundação para a Ciência e a Tecnologia to LRR (grant number EXPL/BIA‐EVL/0131/2021; http://doi.org/10.54499/EXPL/BIA‐EVL/0131/2021) and to CE3C (unit funding: UIDB/00329/2020; https://doi.org/10.54499/UIDB/00329/2020).

## CONFLICT OF INTEREST STATEMENT

The authors have no conflict of interest to declare.

### OPEN RESEARCH BADGES

This article has earned Open Data, Open Materials and Preregistered Research Design badges. Data, materials and the preregistered design and analysis plan are available at https://doi.org/10.5061/dryad.hdr7sqvp9.

## Supporting information


Appendix S1:

## Data Availability

Analyses reported in this article can be reproduced using the data and code that will be publicly available in Dryad upon acceptance. For reviewing purposes, we provide the corresponding private link: https://datadryad.org/stash/share/OCdqzNAQDww5T9e8jtUIUiWmTyptqxkcOAf6nQtc8UY.

## References

[ece311493-bib-0001] Alpedrinha, J. , Rodrigues, R. , Magalhães, S. , & Abbott, J. (2019). The virtues and limitations of exploring the eco‐evolutionary dynamics of sexually selected traits. Oikos, 128, 1381–1389. 10.1111/oik.06573

[ece311493-bib-0002] Bates, D. , Mächler, M. , Bolker, B. , & Walker, S. (2015). Fitting linear mixed‐effects models using lme4. Journal of Statistical Software, 67, 1–48. 10.18637/jss.v067.i01

[ece311493-bib-0003] Bolker, B. M. , Brooks, M. E. , Clark, C. J. , Geange, S. W. , Poulsen, J. R. , Stevens, M. H. H. , & White, J. S. S. (2009). Generalized linear mixed models: A practical guide for ecology and evolution. Trends in Ecology & Evolution, 24, 127–135. 10.1016/j.tree.2008.10.008 19185386

[ece311493-bib-0004] Boulton, R. A. , Zuk, M. , & Shuker, D. M. (2018). An inconvenient truth: The unconsidered benefits of convenience polyandry. Trends in Ecology & Evolution, 33, 904–915. 10.1016/j.tree.2018.10.002 30376988

[ece311493-bib-0005] Bretman, A. , Westmancoat, J. D. , Gage, M. J. G. , & Chapman, T. (2011). Males use multiple, redundant cues to detect mating rivals. Current Biology, 21, 617–622. 10.1016/J.CUB.2011.03.008 21439827

[ece311493-bib-0006] Bro‐Jørgensen, J. (2010). Dynamics of multiple signalling systems: Animal communication in a world in flux. Trends in Ecology & Evolution, 25, 292–300. 10.1016/j.tree.2009.11.003 20022401

[ece311493-bib-0007] Candolin, U. (2003). The use of multiple cues in mate choice. Biological Reviews, 78, 575–595. 10.1017/S1464793103006158 14700392

[ece311493-bib-0008] Candolin, U. (2019). Mate choice in a changing world. Biological Reviews, 94, 1246–1260. 10.1111/BRV.12501 30762277

[ece311493-bib-0009] Clemente, S. H. , Rodrigues, L. R. , Ponce, R. , Varela, S. A. M. , & Magalhães, S. (2016). Incomplete species recognition entails few costs in spider mites, despite first‐male precedence. Behavioral Ecology and Sociobiology, 70, 1161–1170. 10.1007/s00265-016-2124-0

[ece311493-bib-0010] Coss, D. A. , Ryan, M. J. , Page, R. A. , Hunter, K. L. , & Taylor, R. C. (2022). Can you hear/see me? Multisensory integration of signals does not always facilitate mate choice. Behavioral Ecology, 33, 903–911. 10.1093/BEHECO/ARAC061

[ece311493-bib-0011] Crawley, M. J. (2007). The R book. John Wiley & Sons, Ltd.

[ece311493-bib-0012] Dore, A. A. , McDowall, L. , Rouse, J. , Bretman, A. , Gage, M. J. G. , & Chapman, T. (2018). The role of complex cues in social and reproductive plasticity. Behavioral Ecology and Sociobiology, 72(8), 1–15. 10.1007/S00265-018-2539-X PMC6060796 30100665

[ece311493-bib-0013] Fawcett, T. W. , & Frankenhuis, W. E. (2015). Adaptive explanations for sensitive windows in development. Frontiers in Zoology, 12, 1–14. 10.1186/1742-9994-12-S1-S3/TABLES/2 26816521 PMC4722342

[ece311493-bib-0014] Fawcett, T. W. , & Johnstone, R. A. (2003). Optimal assessment of multiple cues. Proceedings of the Royal Society B: Biological Sciences, 270, 1637–1643. 10.1098/rspb.2003.2328 PMC1691413 12908986

[ece311493-bib-0015] Hebets, E. A. , & Papaj, D. R. (2005). Complex signal function: Developing a framework of testable hypotheses. Behavioral Ecology and Sociobiology, 57, 197–214. 10.1007/s00265-004-0865-7

[ece311493-bib-0016] Helle, W. (1967). Fertilization in the two‐spotted spider mite (*Tetranychus urticae*: Acari). Entomologia Experimentalis et Applicata, 10, 103–110. 10.1007/BF00338618

[ece311493-bib-0017] Helle, W. , & Sabelis, M. W. (Eds.). (1985). Spider mites: Their biology, natural enemies and control. Elsevier Science Publishing Company.

[ece311493-bib-0018] Higginson, A. D. , & Reader, T. (2009). Environmental heterogeneity, genotype‐by‐environment interactions and the reliability of sexual traits as indicators of mate quality. Proceedings of the Royal Society B: Biological Sciences, 276, 1153–1159. 10.1098/rspb.2008.1592 PMC2679080 19129106

[ece311493-bib-0019] Jennions, M. , & Petrie, M. (2007). Variation in mate choice and mating preferences: A review of causes and consequences. Biological Reviews, 72, 283–327. 10.1111/j.1469-185X.1997.tb00015.x 9155244

[ece311493-bib-0020] Leonard, A. S. , & Hedrick, A. V. (2009). Single versus multiple cues in mate discrimination by males and females. Animal Behaviour, 77, 151–159. 10.1016/j.anbehav.2008.09.029

[ece311493-bib-0021] Leonard, A. S. , & Hedrick, A. V. (2010). Long‐distance signals influence assessment of close range mating displays in the field cricket, *Gryllus integer* . Biological Journal of the Linnean Society, 100, 856–865. 10.1111/j.1095-8312.2010.01472.x

[ece311493-bib-0022] Macke, E. , Magalhães, S. , Khanh, H. D. T. , Frantz, A. , Facon, B. , & Olivieri, I. (2012). Mating modifies female life history in a haplodiploid spider mite. The American Naturalist, 179, 147–162. 10.1086/665002 22504549

[ece311493-bib-0023] Muñoz, N. E. , & Blumstein, D. T. (2012). Multisensory perception in uncertain environments. Behavioral Ecology, 23, 457–462. 10.1093/BEHECO/ARR220

[ece311493-bib-0024] Nagelkerke, C. J. , & Sabelis, M. W. (1996). Hierarchical levels of spatial structure and their consequences for the evolution of sex allocation in mites and other arthropods. The American Naturalist, 148, 16–39. 10.1086/285909

[ece311493-bib-0025] Oku, K. (2010). Males of the two‐spotted spider mite attempt to copulate with mated females: Effects of double mating on fitness of either sex. Experimental & Applied Acarology, 50, 107–113. 10.1007/s10493-009-9306-7 19760507

[ece311493-bib-0026] Oku, K. (2013). Does female mating history affect mate choice of males in the two‐spotted spider mite *Tetranychus urticae*? Acarologia, 53, 217–220. 10.1051/acarologia/20132090

[ece311493-bib-0027] Oku, K. (2014). Sexual selection and mating behavior in spider mites of the genus *Tetranychus* (Acari: Tetranychidae). Applied Entomology and Zoology, 49, 1–9. 10.1007/s13355-013-0238-7

[ece311493-bib-0028] Penman, D. R. , & Cone, W. W. (1972). Behaviorof male twospotted spider mites in response to quiescent female deutonymphs andto web. Annals of the Entomological Society of America, 65(6), 1289–1293.

[ece311493-bib-0029] Penman, D. R. , & Cone, W. W. (1974). Role of web, tactilestimuli, and female sex pheromone in attraction of male twospotted spider mitesto quiescent female deutonymphs. Annals of the EntomologicalSociety of America, 67(2), 179–182.

[ece311493-bib-0030] Reeve, H. (1989). The evolution of conspecific acceptance thresholds. The American Naturalist, 133, 407–435.

[ece311493-bib-0031] Rodrigues, L. R. , Figueiredo, A. R. T. , Van Leeuwen, T. , Olivieri, I. , & Magalhães, S. (2020). Costs and benefits of multiple mating in a species with first‐male sperm precedence. The Journal of Animal Ecology, 89, 1045–1054. 10.1111/1365-2656.13171 31872443

[ece311493-bib-0032] Rodrigues, L. R. , Figueiredo, A. R. T. , Varela, S. A. M. , Olivieri, I. , & Magalhães, S. (2017). Male spider mites use chemical cues, but not the female mating interval, to choose between mates. Experimental & Applied Acarology, 71, 1–13. 10.1007/s10493-016-0103-9 28040863

[ece311493-bib-0033] Rodrigues, L. R. , & Magalhães, S. (2024). Fake news? The impact of information mismatch in mating behaviour (Dataset). Dryad. 10.5061/dryad.hdr7sqvp9 PMC11255374 39026965

[ece311493-bib-0034] Rodrigues, L. R. , Zélé, F. , Santos, I. , & Magalhães, S. (2022). No evidence for the evolution of mating behavior in spider mites due to *Wolbachia*‐induced cytoplasmic incompatibility. Evolution, 76, 623–635. 10.1111/evo.14429 35092614

[ece311493-bib-0035] Ronald, K. L. , Zhang, X. , Morrison, M. V. , Miller, R. , & Hurley, L. M. (2020). Male mice adjust courtship behavior in response to female multimodal signals. PLoS One, 15, e0229302. 10.1371/journal.pone.0229302 32241020 PMC7117945

[ece311493-bib-0036] Royalty, R. N. , Phelan, P. L. , & Hall, F. R. (1993). Comparative effects of form, colour, and pheromone of twospotted spider mite quiescent deutonymph on male guarding behaviour. Physiological Entomology, 18, 303–316. 10.1111/j.1365-3032.1993.tb00603.x

[ece311493-bib-0037] Rypstra, A. L. , Schlosser, A. M. , Sutton, P. L. , & Persons, M. H. (2009). Multimodal signalling: The relative importance of chemical and visual cues from females to the behaviour of male wolf spiders (Lycosidae). Animal Behaviour, 77, 937–947. 10.1016/J.ANBEHAV.2008.12.026

[ece311493-bib-0038] Satoh, Y. , Yano, S. , & Takafuji, A. (2001). Mating strategy of spider mite, *Tetranychus urticae* (Acari: Tetranychidae) males: Postcopulatory guarding to assure paternity. Applied Entomology and Zoology, 36, 41–45. 10.1303/aez.2001.41

[ece311493-bib-0039] Scharf, H. M. , Suarez, A. V. , Kern Reeve, H. , & Hauber, M. E. (2020). The evolution of conspecific acceptance threshold models. Philosophical Transactions of the Royal Society B, 375, 20190475. 10.1098/rstb.2019.0475 PMC7331004 32420847

[ece311493-bib-0040] Scheuber, H. , Jacoty, A. , & Brinkhof, M. W. G. (2004). Female preference for multiple condition‐dependent components of a sexually selected signal. Proceedings of the Royal Society of London. Series B: Biological Sciences, 271, 2453–2457. 10.1098/rspb.2004.2907 PMC1691884 15590595

[ece311493-bib-0041] Schneeberger, K. , & Taborsky, M. (2020). The role of sensory ecology and cognition in social decisions: Costs of acquiring information matter. Functional Ecology, 34, 302–309. 10.1111/1365-2435.13488

[ece311493-bib-0042] Smith, J. M. (1964). Group selection and kin selection. Nature, 201, 1145–1147. 10.1038/2011145a0

[ece311493-bib-0043] Snook, R. R. (2014). The evolution of polyandry. In D. M. Shuker & L. W. Simmons (Eds.), The evolution of insect mating systems (pp. 159–180). Oxford University Press.

[ece311493-bib-0044] Stoehr, A. M. , & Wojan, E. M. (2016). Multiple cues influence multiple traits in the phenotypically plastic melanization of the cabbage white butterfly. Oecology, 182, 691–701. 10.1007/S00442-016-3694-2/FIGURES/3 27417547

[ece311493-bib-0045] Stoltz, J. A. , McNeil, J. N. , & Andrade, M. C. B. (2007). Males assess chemical signals to discriminate just‐mated females from virgins in redback spiders. Animal Behaviour, 74, 1669–1674. 10.1016/j.anbehav.2007.03.011

[ece311493-bib-0046] Therneau, T. M. (2015). Mixed effects cox models. R Package, 2, 1–21.

[ece311493-bib-0047] Thomas, M. L. (2011). Detection of female mating status using chemical signals and cues. Biological Reviews, 86, 1–13. 10.1111/j.1469-185X.2010.00130.x 20233168

[ece311493-bib-0048] Thornhill, R. , & Alcock, J. (1983). The evolution of insect mating systems. Harvard University Press.

[ece311493-bib-0049] Tibbetts, E. A. , Liu, M. , Laub, E. C. , & Shen, S.‐F. (2020). Complex signals alter recognition accuracy and conspecific acceptance thresholds. Philosophical Transactions of the Royal Society B, 375(1802), 20190482. 10.1098/rstb.2019.0482 PMC7331021 32420854

[ece311493-bib-0050] Yasui, Y. (1994). Adaptive control of copulation duration by males under sperm competition in the mite, *Macrocheles muscaedomesticae* . Experimental & Applied Acarology, 18, 543–554. 10.1007/BF00058937

[ece311493-bib-0051] Zélé, F. , Santos, I. , Olivieri, I. , Weill, M. , Duron, O. , & Magalhães, S. (2018). Endosymbiont diversity and prevalence in herbivorous spider mite populations in South‐Western Europe. FEMS Microbiology Ecology, 94, 1–11. 10.1093/femsec/fiy015 29390142

