# Supplementary material for: Fake news? The impact of information mismatch on mating behaviour
Source: Ecol Evol. 2024 Jul 17;14(7):e11493. doi: 10.1002/ece3.11493 (PMC11255374; doi:10.1002/ece3.11493)
Supplement: Supplementary file 1 — Appendix S1: [file ECE3-14-e11493-s001.docx]

**Supplementary Material**

**Fake news? The impact of information mismatch in mating behaviour**

**Table S1.** Statistical models used in the analysis of reproductive traits.

**Table S2.** Statistical results obtained for male and female behavioural traits and for male survival.

**Table S1. Statistical models used in the analysis of reproductive traits. “**Sample size” corresponds to the number of patches used in each analysis. "Maximal model" gives the complete set of explanatory variables (and their interactions) included in the model (note: “*” represents both the interaction between two explanatory variables and their main effect). "Minimal model" gives the model containing only the explanatory variables and interactions between them that were statistically significant. Variables in round brackets were included as random factors. Square brackets indicate the error structure used (p: poisson, b: binomial; g: gaussian; gt: gaussian transformed). “Substrate cues”: cues left on the patches by virgin or mated females prior to the beginning of the mating sessions; “mating status”: virgin or mated females that were exposed to males in the already impregnated patches; “block: session of mating observations; “order”: the rank of each mating (i.e., the first, second or third mating, etc, of a male). ^a^ excludes replicates in which the duration of a copula was lost; ^b^ excludes four lost replicates.

| **var. of interest** | **response variable** | **Sample size** | **Maximal model** | **Minimal model** | **R subroutine [err struct.]** |
| --- | --- | --- | --- | --- | --- |
| Mating attempts | nr of times a male touched a female with the two front legs and started bending its opisthosoma | 84 | substrate cues*mating status + (block) | substrate cues + (block) | glmer [p] |
| Mating acceptance | cbind (nr female acceptances,  nr female rejections) | 84 | substrate cues * mating status + (block) | substrate cues + mating status + (block) | glmer [b] |
| Mating events | nr matings | 84 | substrate cues * mating status + (block) | substrate cues + mating status + (block) | glmer [p] |
| Copulation duration of first mating | time a male spent copulating in the first mating | 84 | substrate cues * mating status + (block) | substrate cues + mating status + (block) | lmer [g] |
| Copulation duration across events | time a male spent copulating in each mating | 81^a^ | substrate cues * mating status *order + (block) | substrate cues + mating status *order + (block) | lmer [gt] |
| Survival | nr of days a male stayed alive | 80^b^ | substrate cues * mating status + (nr matings) + (block) | substrate cues + mating status + (block) | coxme |

**Table S2. Statistical results obtained for male and female behavioural traits and for male survival.** Df indicates the degrees of freedom. Whenever the significance of the explanatory variables was established using an F-test, the value between brackets indicates the residual degrees of freedom. χ^2^/F provides the χ^2^ or the F value obtained in each analysis, depending on which distribution was used. “Substrate cues”: cues left on the patches by virgin or mated females prior to the beginning of the mating sessions; “mating status”: virgin or mated females that were exposed to males in the already impregnated patches; “order”: the rank of each mating (i.e., the first, second or third mating, etc, of a male). Statistically significant contrasts are represented in bold.

| **Var. of interest** | **Explanatory var.** | **Df (Df res.)** | **χ^2^/F** | **P value** |
| --- | --- | --- | --- | --- |
| Mating attempts | substrate cues x mating status | 1 | 1.104 | 0.293 |
|  | **substrate cues** | **1** | **54.323** | **<0.001** |
|  | mating status | 1 | 2.055 | 0.152 |
| Mating acceptance | substrate cues x mating status | 1 | 2.625 | 0.105 |
|  | **substrate cues** | **1** | **8.553** | **0.004** |
|  | **mating status** | **1** | **64.252** | **<0.001** |
| Mating events | substrate cues x mating status | 1 | 1.274 | 0.259 |
|  | **substrate cues** | **1** | **35.445** | **<0.001** |
|  | **mating status** | **1** | **17.894** | **<0.001** |
| Copulation duration of first mating | substrate cues x mating status | 1 (60) | 0.002 | 0.966 |
|  | **substrate cues** | **1 (61)** | **4.737** | **0.033** |
|  | **mating status** | **1 (61)** | **70.367** | **<0.001** |
| Copulation duration across events | substrate cues x mating status x order | 1 (415.50) | 0.050 | 0.823 |
|  | substrate cues x mating status | 1 (150.24) | 0.386 | 0.536 |
|  | substrate cues x order | 1 (350.60) | 0.849 | 0.358 |
|  | **mating status x order** | **1 (364.06)** | **5.652** | **0.018** |
|  | **substrate cues** | **1 (72.70)** | **4.624** | **0.035** |
|  | **mating status** | **1 (57.36)** | **202.449** | **<0.001** |
|  | **order** | **1 (420.01)** | **166.388** | **<0.001** |
| Survival | substrate cues x mating status | 1 | 0.496 | 0.481 |
|  | nr matings | 1 | 0.051 | 0.446 |
|  | **substrate cues** | **1** | **4.283** | **0.038** |
|  | **mating status** | **1** | **8.774** | **0.003** |
